# Local markets for global health technologies: lessons learned from advancing 6 new products

**DOI:** 10.9745/GHSP-D-13-00131

**Published:** 2014-05-13

**Authors:** Dipika Mathur Matthias, Catharine H Taylor, Debjeet Sen, Mutsumi Metzler

**Affiliations:** a PATH, Seattle, WA, USA; b PATH, Washington, DC, USA. Now with Management Sciences for Health, Arlington, VA, USA; c PATH, Washington, DC, USA

## Abstract

Key components to support local institutional and consumer markets are: supply chain, finance, clinical use, and consumer use. Key lessons learned: (1) Build supply and demand simultaneously. (2) Support a lead organization to drive the introduction process. (3) Plan for scale up from the start. (4) Profitability for the private sector is an absolute.

## INTRODUCTION

Global procurement mechanisms are a major potent force in successful global health programming. Such mechanisms purchase key technologies such as vaccines, antiretroviral drugs, and contraceptives for distribution across many countries. However, many more critical technologies, particularly those affecting maternal and newborn health, require advancement of local markets for ultimate sustainability and public health impact. Unfortunately, many of these markets suffer from a basic dysfunction: the lack of sufficient market incentive to stimulate production and distribution as well as complicated local supply chains and delivery systems to reach those most in need.

A multifaceted approach is needed to address the basic causes of dysfunction in the market for public health commodities.

In this article, we present 6 case studies of technologies recently introduced into developing-country markets (Oxytocin in Uniject, *care*HPV, Helping Babies Breathe, Woman's Condom, Safe Water, and Ultra Rice). We use a market introduction framework as an organizing structure to highlight key elements that may have contributed to varying degrees of success and, when lacking, to certain challenges in these markets. Through these examples, we hope to contribute to the global discussion on best practices for creating healthy markets as the global health community works together to accelerate access to lifesaving technologies.

## MARKET TYPES: GLOBAL, INSTITUTIONAL, AND CONSUMER

To cultivate the market for a given commodity, it is important to first understand key characteristics of that market. Although the boundaries between different market classifications are somewhat permeable, there are generally 3 types of markets for global health technologies:

- **Globally coordinated markets,** where technologies such as vaccines are procured and financed through centralized channels. Typically, in these types of markets a small number of buyers, such as the GAVI Alliance (formerly the Global Alliance for Vaccines and Immunization), the United Nations Children's Fund (UNICEF), and the Global Fund to Fight AIDS, Tuberculosis and Malaria, purchase large volumes of product through pooled procurement at low prices, which are then distributed through relatively well-organized channels.
- **Local institutional markets,** in which national institutions, such as a ministry of health, purchase technologies such as drugs used for obstetric care, whether through their own resources or from donor grants or loans. These markets require strong facility inputs, such as skilled health workers, complex clinical protocols to ensure proper use of commodities, robust logistics systems to ensure commodities and equipment remain continuously available at health facilities, and local health budgets.
- **Consumer markets,** in which a large number of disaggregated consumers buy health goods, such as oral rehydration salts or water filters, for their own use. Although the value proposition may be clearer and the products easier to use than those in institutional markets, these products require purchase by consumers, who often do not consider health-related purchases a high priority. These markets also require significant investments in distribution and marketing by private-sector companies that naturally prefer to serve consumers with higher incomes due to the potential for greater profit margins and more consistent demand.

The crucial role of the latter two, or “local,” markets is amply demonstrated by the work of the UN Commission on Life-Saving Commodities for Women and Children (UNCoLSC), which is leading global efforts to improve access to 13 potentially lifesaving commodities essential to maternal and neonatal health (Table). The Commission has developed key recommendations to address the most critical bottlenecks facing product introduction, from innovation and global market-shaping to local delivery and demand generation.

**Table:** TABLE. Market Classification for UNCoLSC Technologies

**Technology**	**Globally Coordinated Markets**	**Local Institutional Markets**	**Consumer Markets**
**Maternal Health**			
Oxytocin		✓	✓
Misoprostol		✓	✓
Magnesium sulfate		✓	✓
**Newborn Health**			
Antenatal corticosteroids		✓	
Injectable antibiotics		✓	✓
Neonatal resuscitation		✓	
Chlorhexidine		✓	✓
**Family Planning**			
Female condoms	✓	✓	✓
Implants	✓	✓	
Emergency contraception	✓		✓
**Child Health**			
Oral rehydration salts		✓	✓
Amoxicillin		✓	✓
Zinc		✓	✓

Abbreviation: UNCoLSC, UN Commission on Life-Saving Commodities for Women and Children.

Compared with global procurement, both institutional and consumer markets have less defined advocacy and policy pathways and often suffer from lack of donor funding to continue purchasing the product beyond initial introduction pilots, as they do not have large-scale, committed funding mechanisms behind them. Manufacturers are often left with insufficient demand to continue supply, while buyers have limited experience with and understanding of the value of the technology and, hence, have weak motivation to purchase.

In institutional and consumer markets, manufacturers are often left with insufficient demand to continue supply, while buyers have limited experience with and understanding of the value of the technology.

Beyond the lack of financing, there are several additional reasons that these markets have not been effectively established. The framework we offer attempts to capture the broad range of elements required for effective introduction of global health products into local institutional and consumer markets.

## MARKET INTRODUCTION FRAMEWORK

A broad search of the published literature revealed that few market introduction frameworks for global health technologies exist. Most players involved in shaping global health markets rely on informal, unpublished frameworks that generally describe the market problems (such as high price), identify specific causes (such as risk for manufacturers), and then focus on interventions to address the root cause (such as enhancing demand through advanced purchase).^1^ These approaches tend to focus on the traditional market levers of price, volume, information, and quality. They are useful analytical frameworks that can help us identify which market interventions might address the problem most effectively.

However, by defining the market introduction sphere more broadly, we are better able to consider the larger range of causes for success or failure in these markets, and consequently, a broader range of potential interventions. This thinking has been captured in some excellent frameworks addressing the diffusion of health innovations. For example, Atun et al. point out the need to clearly delineate the problem, the willingness of major stakeholders to support adoption, and the readiness of health systems infrastructure.^2^ Free also considers a wide range of factors, such as appropriate design of the technology and engagement of gatekeepers.^3^

By defining the market introduction sphere more broadly, we are better able to consider the larger range of causes for success or failure in these markets.

Our market introduction framework builds on this critical systems-wide thinking; it is a modified version of a framework initially developed by Hozumi et al., through interviews with stakeholders with scale-up experience and through retrospective evaluations of global health interventions advanced by PATH.^4^ This empirically driven framework serves as more of a “how-to” guide than an analytical framework. It focuses on critical market design factors related to the introduction of global health products, assuming that external elements, such as a strong leader and relevance of the product to the global health context, are already in place. (Often, even relevant products with strong champions still fail to reach market.)

Developing, deploying, and scaling up global health technologies is a multistep, iterative process taking many years, starting with research/design and development/validation, through regulatory approval, introduction, and scale up. This framework elaborates only on the introduction phase of this typical value chain for technologies.

Introduction, particularly for institutional and consumer markets, has several elements that must come together to ensure both the availability of the commodity and its effective use. In fact, the introduction phase is often where global health technologies destined for local markets falter. This phase can be broken down into its essential components of both supply and demand, as illustrated by the 4 pathways in the Figure: (1) supply chain, (2) finance, (3) clinical use, and (4) consumer use. The supply chain and finance pathways are relevant to both institutional and consumer markets, while clinical use pertains only to institutional markets and consumer use only to consumer markets. These critical pathways tend to run in parallel.

**FIGURE.**
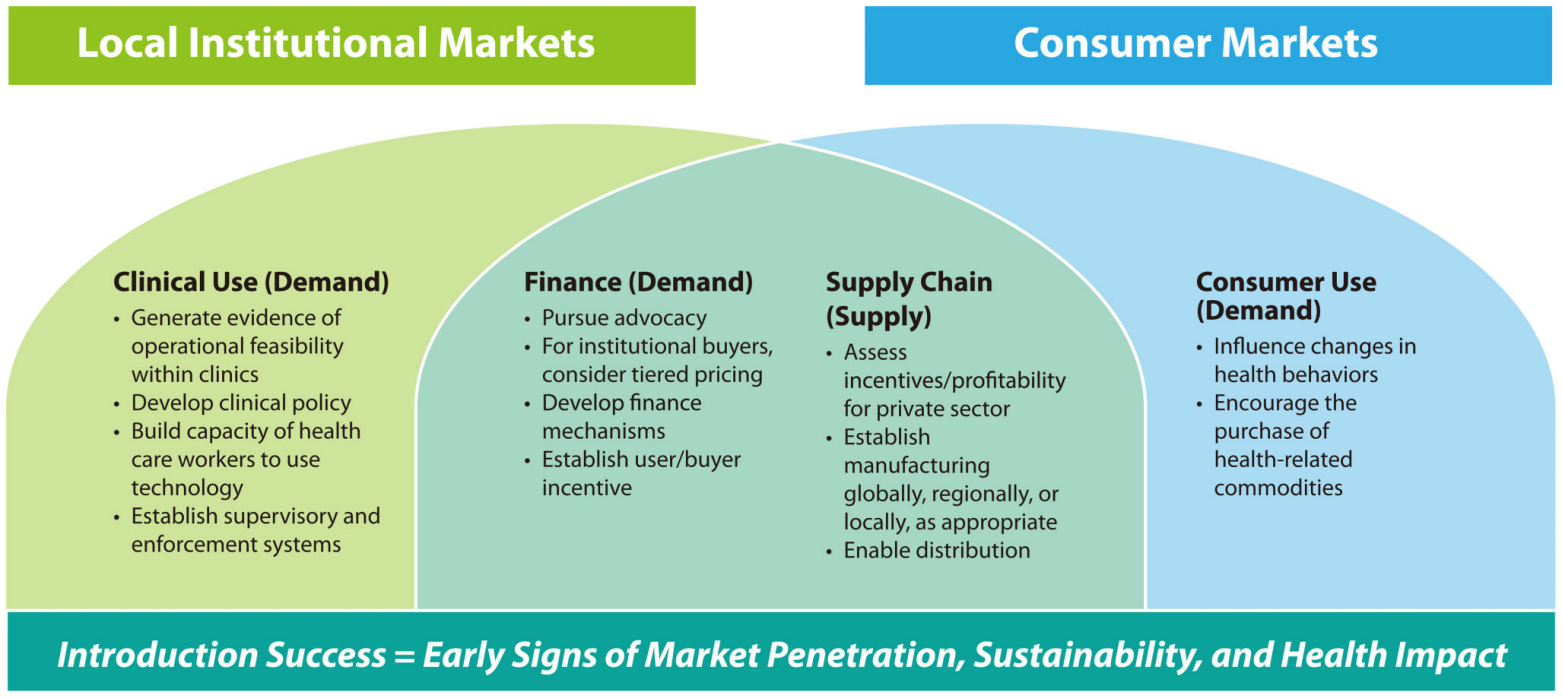
Framework for Introducing Global Health Technologies Into Local Markets

The introduction phase is often where global health technologies destined for local markets falter.

### 1. Supply Chain Pathway

Within the introduction phase, the supply chain pathway in both institutional and consumer markets focuses on conventional supply-chain development. For technologies that are capital-intensive or where quality manufacturing cannot be established locally, a centralized, global manufacturer may be the most effective commercialization strategy. However, as quality improves, technologies have increasingly been licensed to regional or local manufacturers, and appropriate manufacturing equipment needs to be purchased. A formal transfer of the technology is then conducted and manufacturing is validated. Also, distribution channels to appropriate clinical facilities, retail stores, and other outlets need to be established.

### 2. Finance Pathway

The finance pathway focuses on enhancing demand through appropriate financing for the buyers of these technologies. For institutional markets, advocacy is often required to ensure appropriate institutional financing mechanisms, whether through government or private facilities, to support ongoing clinical use of the technology. This requires cultivating multiple champions in the ministries of health and finance to advocate the necessary budget allocations. Tiered pricing strategies, vouchers, and full subsidies may need to be considered by institutional buyers who pass a portion of their costs to distributors, health facilities, or consumers. Consumer products may also require financing, sometimes in the form of low-cost loans, particularly for more costly durable goods such as water filtration systems; low-income consumers often cannot pay the full cost upfront. These financing mechanisms can be provided through microfinance institutions and other businesses focused on consumer finance. Sometimes incentives, such as longer payback periods, are needed for consumers to take advantage of these financing mechanisms.

### 3. Clinical Use Pathway

A third and very important introduction pathway, particularly for institutional markets, is creating demand among providers and institutions by developing the clinical capacity to use the technology within health facilities. This is a rigorous undertaking in itself; it entails generating operational evidence on use of the technology in clinic settings, developing formal clinical policy to guide how the technology would be used appropriately within a clinical protocol, training to ensure that health workers understand the revised clinical guidelines, and establishing enforcement mechanisms, such as supervisory systems, to ensure sustained use. Lack of movement down this pathway has significantly slowed the adoption of many new global health technologies.

A very important introduction pathway is creating demand by developing capacity to use the technology in health facilities.

### 4. Consumer Use Pathway

The demand side for consumer markets usually requires social marketing to influence consumer purchase of products sold through retail channels, which may or may not be partially subsidized. Traditional behavior-change techniques also are used to encourage uptake of consumer products distributed through public and private-sector channels. For instance, village leaders may discuss healthy behaviors with mothers groups; in doing so, they may encourage the use of health products for their family, such as oral rehydration salts to treat diarrhea or soap to prevent infection.

These introduction activities, if carefully orchestrated and pursued in parallel, provide the basis for successful market introduction of global health products. As noted in the Figure, introduction success is achieved through early signs of market penetration, sustainability through public, private, and nongovernmental partners, and, ultimately, health impact in target markets. More significant penetration of these outcomes would indicate achievement of scale. We use this introduction framework in a retrospective analysis of 6 technologies—3 from institutional markets and 3 from consumer markets—each with varying degrees of success and failure. This framework can also serve as a guide for new market introduction efforts.

## CASE STUDIES FROM INSTITUTIONAL MARKETS

### Oxytocin in Uniject* Injection System: Supply Chain and Financing Hinder Market Introduction

Oxytocin in Uniject is an example of an introduction pathway complicated by high costs, leading to difficulty in establishing the supply chain with manufacturers and distribution partners, and, consequently, a financing pathway as well. Thus, the market for Oxytocin in Uniject has not yet been established.

There is a strong public health case for this technology. Hemorrhage is the leading cause of maternal death in low-income countries, responsible for an estimated one-third of such deaths annually,^5^ and postpartum hemorrhage accounts for the majority of obstetric hemorrhage cases.^6^ For prevention of postpartum hemorrhage, oxytocin (10 IU) is the drug of choice, recommended by the World Health Organization (WHO).

Oxytocin is temperature-sensitive and needs to be injected intramuscularly. Its use has generally been restricted to medically trained staff in health facilities. Oxytocin in Uniject was developed to simplify oxytocin administration through a single-use, prefilled device that delivers the correct dose. In deliveries attended by auxiliary health workers, Oxytocin in Uniject provides a feasible alternative for delivery of prophylactic oxytocin.[Fig f02]

**Figure f02:**
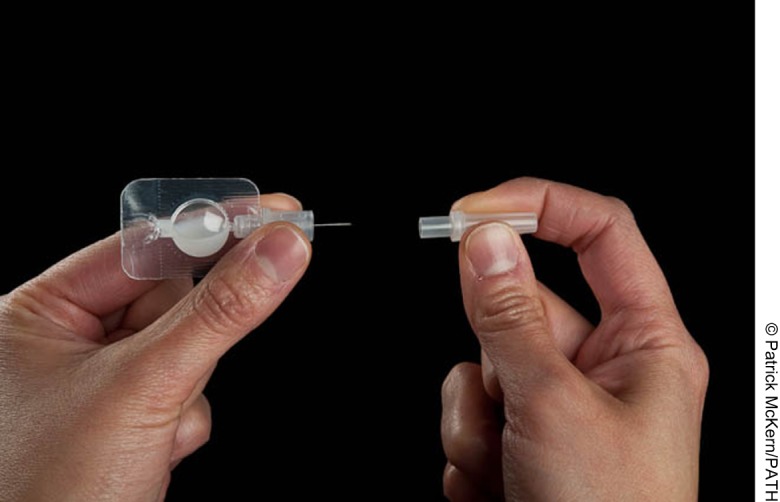
Oxytocin in Uniject delivers oxytocin to prevent postpartum hemorrhage through a single-use, prefilled device.

There is good-quality clinical and operational evidence to support clinical policy permitting use of Oxytocin in Uniject by auxiliary health workers and outside facilities, including effectiveness and feasibility studies.^7^^–^^9^ The product attributes have been tested to ensure that they meet the need at both the community and higher levels of health service. A recent trial in Ghana demonstrated the feasibility at the community level of home delivery for prevention and early treatment.^10^^,^^11^ Thus, the *clinical use* pathway has largely been followed, even though in some countries, medical associations have objected to policies that would allow for task shifting of oxytocin administration to community health care workers. However, the greatest challenge for Oxytocin in Uniject has been in reaching sustainable levels of supply and demand-side financing to catalyze the market.

Cost has been the root problem. Current pricing is more than US$1 per unit, which far exceeds the approximately $0.25 cost of a needle, syringe, and ampoule of oxytocin. To date, buyers are unwilling to pay the premium for oxytocin in a prefilled Uniject device. Furthermore, it is not a needed product at higher levels of the health system, as facilities are well-stocked with needles, syringes, and ampoules of oxytocin. A study in Argentina, however, has demonstrated higher compliance with Oxytocin in Uniject than with conventional administration.^12^

One of the reasons for the higher cost of Oxytocin in Uniject is that it requires pharmaceutical manufacturers to invest significant upfront capital to procure and commission specialized filling and packaging equipment. Donor funding to defray these costs has not been available to the various manufacturers of oxytocin. Additionally, as with any drug in a new form of primary packaging, the pharmaceutical producer would need to apply for and obtain drug regulatory approval in each country where the product would be sold—a long and expensive process that creates further disincentive to supply. This is especially true for those drug and vaccine manufacturers that are in highly competitive generic markets, such as oxytocin, as the investment may not shift in market share of their product.

For those manufacturers in highly competitive markets, an investment in a new delivery mechanism may not shift market share of their product.

We have collaborated with a number of pharmaceutical producers over the past decade to try to establish a sustainable supply of Oxytocin in Uniject. Three producers went as far as producing pilot batches and undertaking the costly stability studies. Due to the lack of clear demand at a price that would be commercially attractive, 2 producers dropped the project before completing development and regulatory approval. One producer stated it had cost close to US$100,000 to conduct the stability studies to WHO's prequalification standards. If that producer could theoretically earn a $0.20 per-dose profit above the manufacturing cost, the company would have to sell 500,000 doses just to recoup the cost of the stability study, which is only one component of the total investment needed to produce and sell Oxytocin in Uniject. The one producer that took Oxytocin in Uniject to market in a number of Latin American countries has not achieved sustainable sales in private-sector channels, and also, as discussed, the institutional markets have not developed. It is not clear whether that producer will keep Oxytocin in Uniject in its product line.

Lack of movement down the finance pathway, also due to price, has further contributed to the difficulty of establishing a sustainable market for the product. Various groups have modeled the cost-effectiveness of Oxytocin in Uniject and have found that its value proposition remains very sensitive to the final price of the product. The current high price has made it difficult to advocate the product's cost-effectiveness, leading to the lack of financial commitment from institutional buyers.

#### Lessons Learned

Regardless of the potential public health benefit and apparent elegance of the approach, an innovation must be extremely affordable relative to the alternatives for institutional uptake to occur. Simply put, we now understand the difficulty of putting an inexpensive product such as oxytocin into a relatively expensive injection device such as Uniject.

Additionally, we have learned that, in the absence of a concerted demand-side intervention, market mechanisms are unlikely to produce sufficient demand to yield significant economies of scale. We had expected the unit price to drop over time as manufacturers achieved economies of scale through high-volume production. We also expected sales in private markets at higher prices to cross-subsidize lower-priced sales in public-sector markets. Neither scenario has materialized.

We have also learned to moderate our expectations of how much drug and vaccine manufacturers in highly competitive generic product markets will invest in regulatory and market introduction of new delivery mechanisms for their products, even with support from the global health community, and especially if demand is unclear.

Finally, we learned that, if pricing does not fall within the target range for institutional buyers as development, manufacturing, and introduction progress, the public health community must consider withdrawing further support sooner in order to redirect energy and resources toward more marketable endeavors that may consequently have more impact. Without market penetration, it follows that sustainability and health impact have yet to be realized from this product.

### *care*HPV†: Lack of Operational Evidence on Clinical Use Slows Pace of Adoption

Despite a clear supply pathway and good evidence of its effectiveness, use of *care*HPV remains limited. The *care*HPV test is an example of how lack of movement down the clinical use pathway, starting with the need for operational evidence of use within facilities, has slowed the pace of adoption.

The *care*HPV test is a screening test that detects the presence of human papillomavirus (HPV), the primary cause of cervical cancer, through cervical or vaginal samples. It was specifically designed for use in developing countries through collaboration between its manufacturer and PATH. Clinical studies, involving more than 20,000 women in 3 countries, have established that *care*HPV is almost twice as accurate as existing screening methods, such as the Pap Smear and Visual Inspection by Acid (VIA), when used as a primary screening method.^13^^,^^14^ Although the cost per test for *care*HPV is higher than for other screening methods, its lower rate of false negatives make it a strong public health tool, considering the high morbidity and mortality if cervical cancer goes undetected. The lower rate of false positives also avoids unnecessary costs associated with follow up. Finally, since *care*HPV can be used with vaginal samples, it can help avoid a pelvic exam, assuming clinical guidelines are in place for self-sampling. However, no country has yet adopted such a practice.

Stakeholder interviews revealed that, in general, clinicians in developing countries are not clear about which screening-to-treatment strategies to use for patients with HPV, since use of HPV tests as the primary screening method is new to both developed and developing countries. WHO guidelines were finally released in 2013, stating that practitioners can go directly from screening with *care*HPV to treatment in low-resource settings, without the need for cytology followed by a diagnostic test such as colposcopy.^15^ It will take time and effort for these guidelines to be mainstreamed into clinical practice.

In addition, policy makers in developing countries are uncertain about how to design the implementation program most appropriate for their local situation as well as about the implications of that approach for their human and financial resources. For instance, since *care*HPV requires processing at a lab, it is unclear how many lab facilities should be enabled within a given district's unique size and capacities in order to maximize both cost efficiencies and appropriate coverage.

#### Lessons Learned

One key lesson learned from the *care*HPV experience is the clear need for operational studies to answer many of these questions, so that appropriate clinical guidelines can be established. This evidence is especially important in the absence of model clinical policies from the developed world that can be followed. In addition, these studies may need to be pursued in several countries to test feasibility in different clinical environments. Simultaneously, financial mechanisms for purchase by ministries of health need to be identified and developed to create a viable and sustainable market. However, since the financial mechanisms may not be established before the evidence is generated and clinical use policies are modified, additional funding from the global health donor community will be required. There is simply insufficient market incentive for the private sector to invest in the operational studies and advocacy required for enabling this market. Without these elements in place, the pace of adoption for *care*HPV will continue to be slow. Again, due to the lack of market penetration at this point, neither sustainability nor health impact has yet been achieved.

Operational evidence is especially important in the absence of clinical use policies from the developed world that can be followed.

### Helping Babies Breathe: Well-Coordinated Supply Chain, Financing, and Clinical Capacity Pathways Provide a Model for Success

The Helping Babies Breathe (HBB) initiative exemplifies the successful introduction of medical equipment into local institutional markets through well-coordinated supply chain, financing, and clinical use pathways. HBB's success is attributable to the U.S. Agency for International Development's (USAID) strong Global Development Alliances (GDAs) model, as well as to the commitment of Laerdal, the key equipment supplier and a founding member of HBB, to saving the lives of newborns in low-resource countries. As such, HBB took the role of a “backbone organization” to orchestrate the various actors and activities along the various introduction pathways.

As a GDA funded by USAID, HBB now includes 20 partners from the private and public sectors. In 2010, HBB began offering evidence-based training and high-quality, affordable neonatal resuscitation equipment to birth attendants in developing countries. As of November 2013, 60 countries had implemented the HBB curriculum, of which 18 have national plans coordinated by governments. In the 60 countries, approximately 130,000 health care providers were trained. In addition, HBB supplied 120,000 bag-and-mask resuscitation devices, 150,000 suction bulbs, and 50,000 simulators on a not-for-profit basis, and donated another 4,500 HBB training kits.

Laerdal supplies high-quality resuscitation equipment at reasonable cost to HBB partners that support the program. Laerdal has also donated simulators and other equipment to enhance use and spark further demand. Additionally, HBB's master training programs, a critical element of the clinical use pathway, provides health care personnel with important operational experience with resuscitation equipment. In selected developing countries, Laerdal also offers grants to support implementation of the training program as well as guidelines on purchasing and maintaining equipment. The training not only increases competencies of birth attendants but also meets their latent need for higher-quality resuscitation equipment. This is important groundwork for creating sustainable, institutional demand from within public-sector facilities.

#### Lessons Learned

Clearly, HBB is a model to follow. HBB has stimulated the supply side of the market through affordable equipment and the demand side through training and implementation grants. In turn, this has created a viable market, maintaining engagement of the many partners and stakeholders involved. While HBB still needs to shift from being mostly donor-driven to country-driven, the initiative's effective coordination role and the demand it has helped to establish by normalizing use of these products in clinic settings bodes well for adoption and scale up of this program by public health institutions in developing countries. It is likely that the role of the market coordinator is an underused mechanism in the uptake of global health products. This example demonstrates clear market penetration, with sustainability now emerging as HBB training guidelines are adopted by various ministries of health and incorporated into global health projects related to newborn resuscitation. Significant health impact is likely to follow.

## CASE STUDIES FROM CONSUMER MARKETS

### Woman's Condom: Lack of Coordination Between Supply and Demand Limit Market Penetration

The Woman's Condom exemplifies the challenge of trying to establish a sustainable supply and distribution chain for a new product when it is not coordinated with an equally well-resourced, demand-generation effort.

The Woman's Condom is a new female condom whose design and early-stage validation was funded initially by USAID to expand contraceptive options for women in developing countries. PATH and its research partners employed a user-centered design process to develop a product that has proved to be highly acceptable.^16^^–^^19^ User acceptability has been a key barrier to use of other female condom products.[Fig f03]

**Figure f03:**
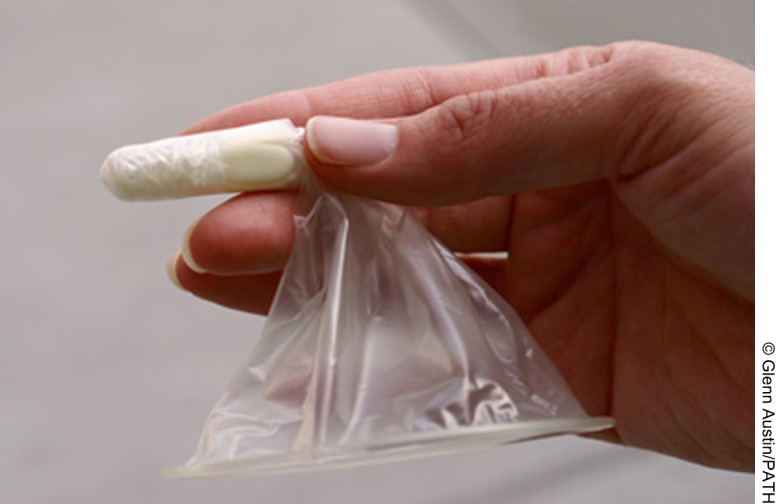
The Woman's Condom employs a user-centered design process to improve acceptability.

Female condoms are primarily a consumer product, in which the decision to purchase and use the product lies with the end user. However, during the first 20 years of introduction, female condom purchasers largely have been bilateral and multilateral institutions, such as USAID, the United Nations Population Fund (UNFPA), and ministries of health, that distribute condoms through government-sponsored programs at free or highly subsidized rates. Although this is a legitimate strategy for introducing a new product class in the absence of robust funding to support demand generation, it has not allowed female condoms to become established as a consumer class product. Manufacturers have focused more attention on sales to institutional buyers, and while new female condom products, such as the Woman's Condom, are helping to enlarge and differentiate the class, consumers are still conditioned to receiving the product for free.

The absence of sustained demand generation is not the only issue. Female condoms are more costly than male condoms, which further reduces demand from both institutional and consumer buyers. For instance, in 2009, donors purchased 71 male condoms for every 1 female condom.^20^

To address these issues, PATH and its manufacturing partner, Dahua Medical Apparatus Company of Shanghai, China, have established a partnership to build a sustainable supply chain for the Woman's Condom, which leverages both public-sector and commercial retail channels. The market penetration strategy is to first generate revenue from private-sector sales to help cover Dahua's costs, thus allowing the company to offer more affordable pricing to low-resources settings served by the public sector in the longer term.

The Woman's Condom has received South Africa Bureau of Standards certification marking (2013), Shanghai Food and Drug Administration approval (2011), and CE (*Conformité Européene*, or European Conformity) marking (2010), which allow for distribution and marketing in private-sector retail channels in South Africa, China, and Europe, respectively. Early introduction efforts in China and South Africa are underway but have already faced challenges. One significant challenge is finding in-country distribution partners willing to invest in the extensive marketing and sales required to generate demand for an unfamiliar product. The cross-subsidy model requires sufficient sales in the commercial sector to cover the costs not recouped through lower prices offered to the public sector. However, due to the investment involved, the revenue from commercial sales is difficult to generate. In China, for example, public-sector sales have exceeded commercial sales, forcing Dahua to sell the product below costs at this time. This situation further reduces Dahua's ability and incentive to invest in the marketing necessary to cultivate private-sector markets.

#### Lessons Learned

A key lesson is the need to pursue aggressive demand-generation efforts simultaneously while establishing supply and distribution chains in order to create strong incentives for actors along the supply chain to engage. Several demand-side elements have been built into the project, including market research with women in China and South Africa, market tests to target consumer markets, and advocacy with the governments in both countries to create a supportive policy environment for female condoms more generally. However, the investment and scale of the demand generation effort has likely been underappreciated. In fact, no real social marketing campaign has yet been launched in either country. Due to the inherent risks involved for manufacturers and distributors, a more significant investment by the donor community may be warranted in order to catalyze this market. Similar to other examples, without market penetration, sustainability and health impact have yet to be achieved with this product.

A significant investment in demand generation is needed for new classes of consumer products.

### Safe Water: Coordinated Supply, Financing, and Consumer Demand Demonstrate Viability of Market

Our Safe Water Project has experimented with several pilot strategies for developing the retail market to sell household water filters to consumers at the base of the economic pyramid. The most successful strategies have involved tight coordination between supply, availability of financing, and consumer demand generation efforts for enhancing the perceived value of the product.

A pilot study in Madhya Pradesh, India, offered consumers free water filters, manufactured by PATH's commercial partner, Hindustan Unilever Limited (HUL), in an effort to draw them into the market. Although 44% of these consumers later took a loan for 2 replacement cartridges from our microfinance partner, Spandana Sphoorty Financial Limited, 35% of consumers reported gifting or selling the water filter. For those who used the product, current and consistent use dropped to 19.1% after 6 months, and then eventually to zero at 10 months.^21^ Since the water filter was a “giveaway,” neither the salespeople nor con-sumers appreciated its value. Therefore, salespeople did not have an incentive to make a strong sales pitch.

In contrast, in Tamil Nadu (Erode), India, the same water filter was offered to consumers without a subsidy. After 10 months, 21% of consumers reported currently using the filters,^20^ suggesting that the strategy succeeded in attracting consumers who valued the product and decided to try it based on their perception of its merits.[Fig f04]

**Figure f04:**
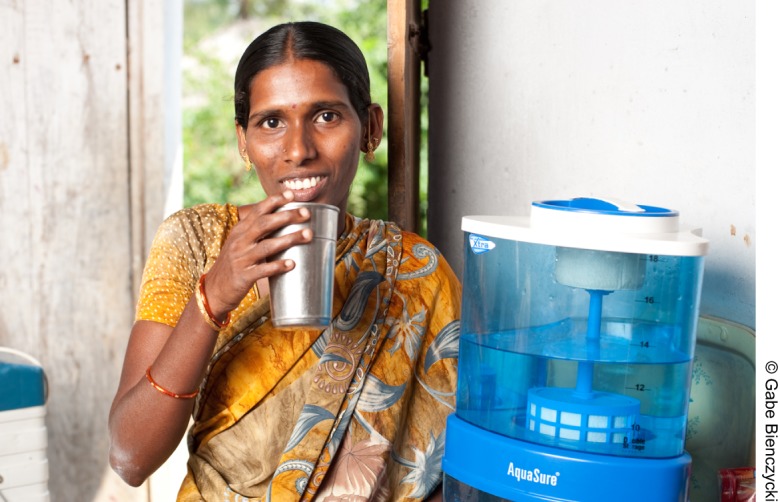
A woman in India uses a household water filter.

The strongest results were observed in Cambodia, where a water filter (Super Tunsai) manufactured by Hydrologic Social Enterprise was paired with a loan scheme offered by the microfinance institution VisionFund. The original Tunsai water filter was not perceived as desirable because of its very basic design and large subsidies to support its use. We worked with Hydrologic to redesign the original Tunsai filter to make it more appealing to customers—both from an aesthetic and functional point of view. The redesigned Super Tunsai filter was then marketed as a desirable product targeting consumers' aspirations toward attaining middle-class status. In spite of the fact that the Super Tunsai cost twice as much as the original Tunsai filter, customers were willing to take full loans from VisionFund to purchase the product. After 10 months, 39% of consumers reported currently using the filter.^21^

After marketing the redesigned Super Tunsai water filter toward consumers' aspirations to attain middle-class status, use improved among consumers.

#### Lessons Learned

Pricing at full retail value is preferable to subsidies, as it attracts more committed consumers and avoids undermining the value of the product, as demonstrated in the Tamil Nadu pilot. We have observed an even stronger effect in Cambodia when this strategy was complemented with social marketing around desirability and affordable replacement cartridge costs. In fact, Hydrologic's replacement cartridges are approximately US$5–$10 per year compared with $25 for the HUL filter used in India. Hydrologic and VisionFund are now scaling up their winning strategy to 11 provinces in Cambodia. Additionally, PATH is using the best practices from its water filter pilots to launch new programs, including a plan to make latrines available in Cambodia through similar schemes, as well as cook stoves, solar lamps, and insecticide-treated bed nets in other markets. The Cambodia pilot, in particular, has demonstrated reasonable market penetration, while signs of sustainability and health impact are emerging.

### Ultra Rice Fortification Technology‡: Sustainability Plans for Both Supply and Demand Drive Scale Up

Efforts to introduce Ultra Rice in Brazil show how creating pillars of sustainability on both the supply and demand sides of the market can enable replication and scale up by local organizations. While PATH's Ultra Rice technology has a 15-year history of fits and starts, the approach in Brazil has created the knowledge and understanding of how to achieve sustainability before the end of each country-specific funding stream under the umbrella project.

The Ultra Rice technology is a formulation and method for creating reconstituted rice grains packed with micronutrients such as iron, thiamin, zinc, and folic acid. When blended with conventional rice, typically at a 1:100 ratio, the resulting fortified rice can provide up to 50% of the recommended daily intake of a range of micronutrients. This helps bridge dietary gaps in micronutrient intake, especially among populations for whom rice constitutes a large portion of their caloric intake.[Fig f05]

**Figure f05:**
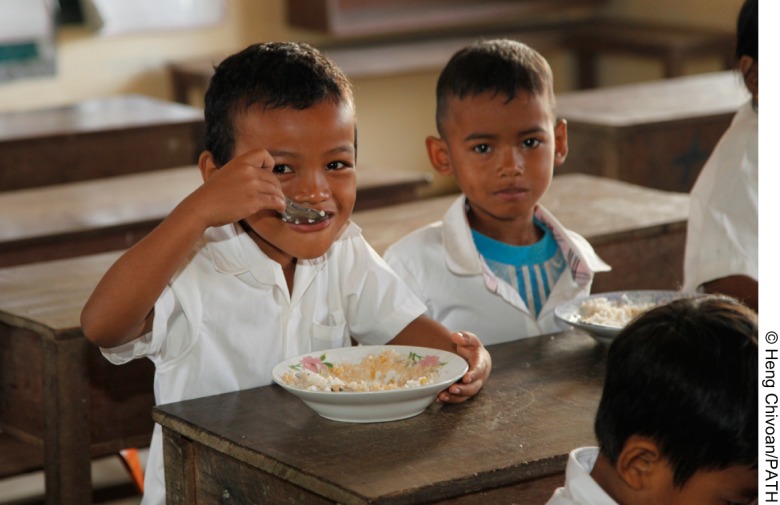
School boys in Cambodia enjoy rice fortified with the Ultra Rice technology to bridge dietary gaps in micronutrients.

In Brazil, the market introduction process for fortified rice started by transferring the technology to local companies, including Urbano Agroindustrial, one of the nation's largest rice millers, and Adorella Aliementos, a medium-sized pasta manufacturer. These companies continue to produce fortified rice kernels, which will not only be incorporated into their own fortified rice products but, as an obligation under PATH's license, also be sold to other millers in order to create a competitive market. Additionally, we have directly licensed the Ultra Rice technology to a preeminent agribusiness university, the Federal University of Viçosa, giving that institution both the right and the obligation to license the technology to additional manufacturers in the future as the market grows. Establishing a small subset of current suppliers as well as an engine for further growth bodes well for supply-side sustainability.

On the demand side, instead of marketing the product primarily to lower-income market segments, which suffer disproportionately from the consequences of micronutrient malnutrition, we are targeting the widest possible range of consumers. In order to establish consumer confidence, the social marketing campaign is built around a quality seal on the packaged rice, conveying aspiration for the product. The Federal University of Viçosa and the national rice millers association, Abiarroz, verify the quality of the fortified grains and homogeneity of their blend into traditional rice. This process is open to all rice brands, including those typically purchased by consumers with lower incomes. While millers of both higher- and medium-price rice brands have led the way, lower-priced brands are likely to move through the verification process in due course, thus leveling the playing field with respect to fortified rice quality. This marketing strategy avoids any stigma that might develop if only the lower-cost brands were fortified and has already reached hundreds of thousands of consumers in the first 6 months since the launch of fortified rice in commercial retail markets.

Additionally, to further enhance demand, we have produced marketing materials for our partners and have negotiated rights to use cartoon characters owned by Turma da Monica (a local “Disney equivalent”), which are well-recognized by Brazilians. Prior to the end of the project, we plan to have these rights transferred to Abiarroz, so that the cartoon characters continue to be used as ambassadors to the product in order to stimulate demand. Also, we have enlisted a number of well-known and highly respected supporters, ranging from noted journalists to well-recognized health experts, who have now established micronutrient malnutrition as one of their social causes.

#### Lessons Learned

Although not all elements of this market penetration strategy can be replicated across rice markets in Africa and Asia, we have learned the value of closely coordinating the various introduction pathways as well as building sustainability into the product introduction plans from the outset. Donor-supported projects that are designed to stimulate global health markets are most successful when engines of further growth for both supply and demand are established well before project funding expires. In other words, planning for sustainability should commence at the start of the project, not after the pilot project has launched and tested the product. In Brazil, planning for sustainability required identifying the array of local organizations that can take ownership of the product and laying out the market development process well before PATH and partners exit. This example demonstrates early signs of significant market penetration and sustainability, although health impact in the target market may be achieved at a slower pace as fortified rice gains further traction in the Brazilian market.

Planning for sustainability should begin at the start of the project.

## SUMMARY

While the global health community has decades of experience bringing technologies, such as vaccines, bed nets, antiretroviral therapies, and malaria drugs, to scale through global procurement mechanisms, we are only now reflecting more systematically on our experience in developing local institutional and consumer markets, especially in light of UNCoLSC's focus on these types of commodities. Global procurement markets require complex coordination among WHO, the GAVI Alliance, UNICEF, and other multilateral institutions to organize purchase and distribution at scale; however, the global health community wrestles even more with local market introduction. The lack of incentives on both demand and supply sides of the market creates natural dysfunction, but our accumulated experience can serve as guideposts as we navigate these challenges.

Among the salient lessons learned from these 6 case studies is the need to design more effective projects (Box). Sometimes, our failure to scale is a failure of design; we either do not recognize all the critical supply and demand elements that need to be pursued or assume other players in the market ecosystem will take them on. One critical design consideration is the need to **build supply and demand simultaneously,** not only to avoid a fatal imbalance, but also to ensure that all relevant demand-side pathways are included in the technology introduction plan, as captured in our framework.

**BOX. Lessons Learned From Introducing Global Technologies Into Local Markets**

- Build supply and demand simultaneously.
- Consider the need for one organization to lead, oversee, and coordinate the entire market introduction activity.
- From the start, have a strong vision and intention to reach full scale through bold, holistic project concepts.
- Pay strong attention to the incentives and profitability of the private sector involved.

Local market introduction of global health products can also benefit from a **lead organization**. Too often in the public health arena, each player, whether a nongovernmental organization (NGO), government, or private-sector firm, supports only a few elements of the required market-development activity, such as generating evidence of operational feasibility or pursuing advocacy for policy change. These small steps are vital elements on the critical path toward scale. Generally, however, no one organization takes on the important role of driving the market introduction process forward—often a necessity in these largely dysfunctional markets. The lack of such a lead is a natural consequence of the piecemeal nature of donor funding and the niche competencies that NGOs have developed to win grants. Few general contractors exist in global health markets. Consequently, we often have ample evidence of potential impact, several interested manufacturers, and strong data to support advocacy, but, despite all this, many of these markets have failed to flourish. A lead organization provides the natural base from which to plan and execute activities along all 4 of the introduction pathways captured in our framework.

Market orchestration also requires a **strong vision and intention to reach scale**—a goal that needs to be built into the project design from its inception and that is embodied within the set of supply and demand-side pathways captured in the framework. Projects driving toward sustainability within their life span create stronger and faster impact. The project nature of donor funding is a cumbersome mechanism for developing local markets. We need to develop stronger models that more closely mirror the required process for reaching scale, such as longer grant periods, flexibility to adjust funding levels to changing market needs, and more holistic project designs.

Finally, but most importantly, we need to pay stronger attention to the **incentives for profitability** of the private sector, which will continue to play an increasingly important role in public health. This is a key element of the supply-side pathway described in the framework. For new technologies, the private sector is often faced with a cost structure that requires pricing above status quo alternatives and uncertain demand due to such issues as the lack of clear distribution channels, clinical policies, and value proposition for the buyer. This most often leads to significant risk and, consequently, an underinvestment in market development by the private sector. While the global health community has developed some effective risk-reduction strategies for products entering global procurement markets, such as advanced market commitments for vaccine development, expedited regulatory pathways for orphan drugs, and graduated copays for vaccine purchases by ministries of health, we need to develop similar, enabling innovations for those products entering local markets. We also need to better analyze the upfront risks to assess whether there would be natural incentives to both supply and adoption of the technology once the risks are sufficiently reduced through an infusion of grant funding. Effective market introduction is paramount and the only way to achieve sustainability.

## CONCLUSION

Our experience underscores the widespread understanding that there is no universally applicable market introduction strategy for products of global health importance. The framework introduced in this article is a starting point, reflecting the general elements of market introduction that may be emphasized differently for each unique product, market, and cultural context. We hope that others continue to build upon the framework with additional insights gained from their experiences. Such empirically-driven frameworks may help minimize our missteps in design, which can be costly and time-consuming and can stymie our collective effort to create health impact in the most vulnerable groups. As a global health community, we need to continue sharing our rich experiences in developing markets for global health products to ensure that global initiatives, such as UNCoLSC, with so many important products destined for local institutional and consumer markets, maximize their potential to save lives.

## Footnotes

* Uniject is a trademark of BD.

† *care*HPV is a trademark of QIAGEN.

‡ Ultra Rice is a registered U.S. trademark of Bon Dente International, Inc.
